# Full-Scale Fatigue Testing of a Wind Turbine Blade in Flapwise Direction and Examining the Effect of Crack Propagation on the Blade Performance

**DOI:** 10.3390/ma10101152

**Published:** 2017-10-03

**Authors:** Othman Al-Khudairi, Homayoun Hadavinia, Christian Little, Gavin Gillmore, Peter Greaves, Kirsten Dyer

**Affiliations:** 1 School of Engineering, Kingston University, London SW15 3DW, UK; oal-khudairi@hotmail.co.uk (O.A.); g.gillmore@kingston.ac.uk (G.G.); 2 Offshore Renewable Energy Catapult (ORE), Blyth NE24 1LZ, UK; Christian.Little@newcastle.ac.uk (C.L.); peter.greaves@ore.catapult.org.uk (P.G.)

**Keywords:** wind turbine, wind blades, flapwise fatigue test, blade modal testing, glass fibre polymer composite

## Abstract

In this paper, the sensitivity of the structural integrity of wind turbine blades to debonding of the shear web from the spar cap was investigated. In this regard, modal analysis, static and fatigue testing were performed on a 45.7 m blade for three states of the blade: (i) as received blade (ii) when a crack of 200 mm was introduced between the web and the spar cap and (iii) when the crack was extended to 1000 mm. Calibration pull-tests for all three states of the blade were performed to obtain the strain-bending moment relationship of the blade according to the estimated target bending moment (BM) which the blade is expected to experience in its service life. The resultant data was used to apply appropriate load in the fatigue tests. The blade natural frequencies in flapwise and edgewise directions over a range of frequency domain were found by modal testing for all three states of the blade. The blade first natural frequency for each state was used for the flapwise fatigue tests. These were performed in accordance with technical specification IEC TS 61400-23. The fatigue results showed that, for a 200 mm crack between the web and spar cap at 9 m from the blade root, the crack did not propagate at 50% of the target BM up to 62,110 cycles. However, when the load was increased to 70% of target BM, some damages were detected on the pressure side of the blade. When the 200 mm crack was extended to 1000 mm, the crack began to propagate when the applied load exceeded 100% of target BM and the blade experienced delaminations, adhesive joint failure, compression failure and sandwich core failure.

## 1. Introduction

One of the main recurring problems within wind turbine blades is fatigue of the blade, especially as the magnitude of the blade size is increasing. The blades are subjected to a highly irregular loading condition caused by turbulent wind flow, gravity, and inertial loading during accelerating or decelerating of the turbine. A wind turbine blade is predominantly loaded in the flapwise and edgewise directions. During operation, the source of flapwise loads are mainly aerodynamic, while the edgewise loads are mainly caused by gravity. The aerodynamic loading is at maximum when the blade position is at 12 O’clock due to higher wind velocity from wind shear, and a minimum when the blade is at 6 O’clock when the blade passes through stagnant air in front of the turbine tower. A modern wind turbine is designed to last 20–30 years almost unattended; hence, a comprehensive testing of the blade is mandatory. Details of the blade testing, inspecting and monitoring procedures can be found in [1,2].

The flapwise loads are carried by the main spar (or girders) while edgewise loads are taken partially by spar and partially, if present, by reinforcement in the leading and trailing edges. Shear webs are located between the two spar caps and provide shear strength to the blade. They are usually made from sandwich structure with bi-axial fibre laminate skins and a central core. The rest of the blade is usually constructed from multi-axial skin material and sandwich structure from glass fibre reinforced polymer (GFRP) composite layers, with a core from foam or balsa wood, ensuring low mass, resistance to torsion and buckling. Characterisation of GFRP materials prior to using in the blade is necessary and the procedure for determining delamination toughness and fatigue performance of GFRP have been reported elsewhere [3,4,5].

As part of the certification procedure, all wind turbine blades are subjected to static, fatigue and failure tests in order to ensure that the produced wind turbine blade fulfill the actual design requirements. Blade manufactures are required to carry out static and fatigue tests of full-scale blades prior to their deployment for commercial use according to international standards [6,7,8,9] and guidelines [10,11]. The static test applies the appropriate extreme load in all directions, i.e. leading edge, trailing edge, suction side and pressure side. The whole static test is then repeated after the fatigue test to ensure that the blade can handle extreme loads after it has been subjected to high cyclic loading. For application of new materials, or other significant changes in the structural design of the blade, a failure test (also called crash test) may be necessary in addition to the static and fatigue tests. After a crash test the blade is cut open at the point of fracture, and the fracture surfaces are examined in detail to find out the root cause of failure. The design may be revised after the forensic analysis of the fracture surfaces.

Yang et al. [12] have done blade crash testing under flapwise loading on a 40 m long blade made from E-glass/epoxy composite materials. They evaluated the structural response of the blade during loading and after crash they correlated experimental data with numerical modelling. They showed the blade sections subjected to bending moments can suffer a non-linearity in their bending response and may crush from the rotated compressive or tensile forces due to the curvature that arises from bending. Kühlmeier [13] showed an interlaminar shear failure originated from local bending in the shell due to an initial geometric imperfection could trigger a progressive collapse of the wind turbine blade section.

Lee and Park [14] tested a 48.3 m wind turbine blade to find the residual strength of the blade subjected to initial static and then fatigue tests. In the positive flapwise direction (from the pressure-side shell to the suction-side shell), the blade was able to sustain the most severe load, but when a static load was applied in the opposite direction the blade collapsed when subjected to 70% of the maximum target load, equivalent to 50% of the most severe load in the positive flapwise direction. Based on the test results and the fracture patterns at the blade’s broken section, they suggested a modified laminate lay-up from (±45/0/core/±45/0) to (±45/0/core/0/±45). Of course this modification makes the laminate symmetric and as a result the coupling stiffness matrix of the laminate, **B** matrix, becomes zero and extension and bending becomes decoupled.

Jensen et al. [15] tested a 34 m wind turbine blade until its structural collapse. The reported failure mechanism was debonding of the outer shell skin followed by delamination buckling. It was noted that the non-linear distortion was caused by the crushing pressure derived from the Brazier effect. The Brazier pressure, where the flexural starts to decrease due to ovalisation of the blade structure, has a significant impact on the design of new blades. They optimized a box girder and showed the importance of including Brazier pressure in the design process for wind turbine blades.

For very long blades, it has been found that buckling failure becomes more important than tip deflection and even fatigue [13]. In blades for safety against buckling a 1.634 safety factor is recommended, meaning that the structure must be capable of withstanding 1.634 times the worst case buckling condition. Cox and Echtermeyer [16] studied the effect of changing the fibre orientations of the less stiff, off-axis glass fibre plies on increasing the critical buckling load of a 70 m carbon/glass hybrid wind turbine blade from nonlinear finite element analysis. They showed the orientation of the stability plies influences the onset of the Brazier effect, which in turn affected blade stability and buckling failure location. They achieved a maximum increase of 8% in critical buckling load by changing the orientation of the stability plies from ±45° as specified in the reference blade to ±70° while both blade weight and laminate thickness remained constant.

Blade root failure can result in the blade being pulled out from its hub during operation. Lee et al. [17] reported delamination failure at the blade root during full-scale fatigue testing of a 3 MW wind turbine blade with length and weight of 56 m and 14.5 ton, respectively. They noticed that the bumping motions of the blade shell redistribute the load at the blade root, resulting in the alleviation of stresses in some locations and the increase of stresses in other locations. They carried out finite element analysis, and showed that in a slender and large wind turbine blade, the actual load distribution at the root is very different from that calculated using assumptions that the blade root has enough stiffness to be modelled as a bending of a hollow circular cylinder.

The fatigue life of the blade can be improved by embedding secondary nanoreinforcements in the polymer matrix. Mishnaevsky and Dai [18] used computational modelling and showed that secondary nanoreinforcement can drastically increase the fatigue lifetime of composites. Many other researchers have shown experimentally that the toughness of the resin can be substantially improved by adding nanoparticles to the polymer matrix [19]. Recently by addition of 0.25 wt % of plasma functionalised graphene nanoparticles to epoxy, the fracture toughness has been increased by more than 50% [20]. Using these new advanced resins will improve a broad range of other physical and mechanical properties of the fibre reinforced composite as well at relatively low cost, a promising material for improving the fatigue damage tolerance of wind turbine blades.

**Figure 1:**
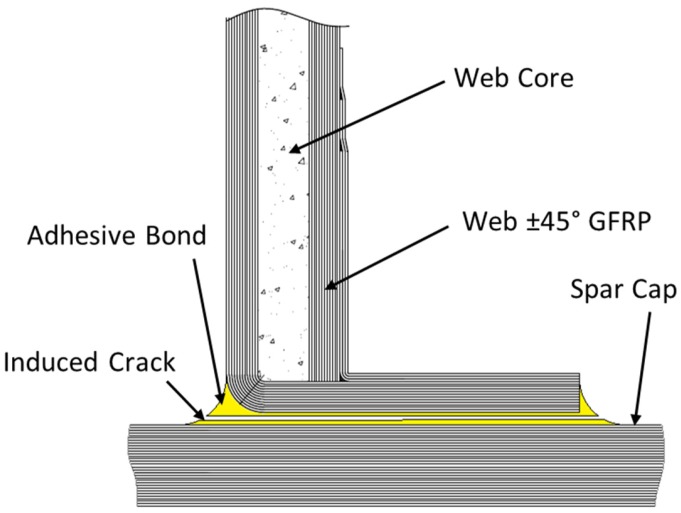
Schematic of induced crack location.

In this paper, a step-by-step full-scale fatigue test of a wind turbine blade is discussed. After the fatigue testing of as received wind turbine blade, the sensitivity of the blade to damage, by manually debonding the shear web from spar cap as shown in Figure 1, has been investigated. The steps of the testing included modal analysis, static, and fatigue testing of a 45.7 m blade in three states of the blade: (i) as received blade (ii) when a crack of 200 mm was introduced between the web and the spar cap and (iii) when the crack was extended to 1000 mm length. As no design data for the blade was available, the location of the crack was chosen to be at a point on the blade which is usually structurally challenging (around max chord) and where the bond line was relatively easy to access.

The calibration pull-tests for all three states of the blade were performed to obtain the strain-bending moment (BM) relationship of the blade according to the target BM which the blade is expected to experience in its service life. The data was used for applying appropriate loads in the fatigue tests. Then the blade natural frequencies in both the flapwise and edgewise directions over a frequency domain range were found by modal testing using hammer impact for all three states of the blade. The IEC 61400-23 standard [6] has then been followed for full-scale blade fatigue testing. Finally, the states of the crack and accompanying damage for all states of the blade have been identified.

## 2. Experimental Studies

### 2.1. Full-Scale Testing Platform

Offshore Renewable Energy (ORE) Catapult has purpose built testing facilities to replicate the harsh fatigue loading which turbine blades experience, within the confines of a test hall. Full-scale blade tests were performed in ORE Catapult 100 m blade test facility capable of static, fatigue, natural frequency testing and for locating the blade centre of gravity.

### 2.2. The Blade Specification

**Figure 2:**
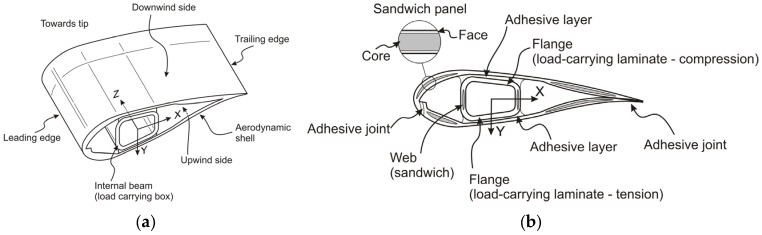
Blade design with a load-carrying box girder [23]. (**a**) The main elements of a wind turbine blade and (**b**) Nomenclature of the different blade construction elements.

The length of the blade used in this study was 45.7 m, which was supplied by the ORE Catapult. The blade is mainly made from glass fibre reinforced plastic (GFRP) composite laminates. In the structure of the blade plies with 0° fibres are used for increasing flapwise bending stiffness while plies with ±45° fibres are used for increasing torsional stiffness and contribute to buckling resistance, along with the 0° plies [21,22]. The detail of terminology for a typical blade is shown in Figure 2.

The blade was initially inspected and some damages and hair line cracks were found on the shell skin on the pressure side at a distance of approximately 33 m from the blade root. Some delamination was also detected at 2 m from the root on the TE.

The blade was subjected to fatigue loading and growth of existing and/or formation of new cracks/damage was monitored using the strain gauges attached to the internal and external blade surfaces, as well as crack visualization. A simple tap test was also done to identify the propagation of delaminations, while acoustic emission sensors were fitted to correlate against visual observations.

### 2.3. Strain Gauge Sensors

Thirty strain gauges were attached to the outside of the blade on the pressure and suction sides, as well as inside the blade on the shear web, before testing.

Table 1 shows the position and distance of strain gauges from the blade root. The applied strain gauges were polyurethane high fatigue encapsulated 350 Ω nominal-resistance strain gauge. The strain data is used to determine the applied BM, and also for detection of any crack propagation.

### 2.4. Accelerometers

During modal testing, five accelerometers were mounted on brackets, which are in turn were hot-glued to the blade using a two-part Araldite 2052 epoxy adhesive (Huntsman, UK). These were used in modal tests repeated for all three states of the blade. Table 2 summarizes the accelerometer locations along the blade.

## 3. Testing Procedure 

### 3.1. Calibration Pull-Test and Position of the Saddles

A calibration pull-test is required to obtain the relationship between the applied load and the bending moment at root. This relationship is used for extrapolation to find the required load to achieve the target BM.

**Figure 3:**
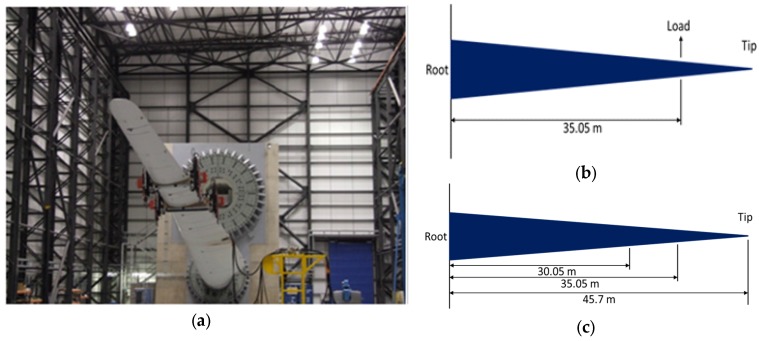
(**a**) 47.5 m blade attached to Hub Centre 1; (**b**) position of applied load point in calibration test and (**c**) the positions of the saddles on the blade for fatigue tests.

Full-scale blade calibration pull-tests were performed for all three states of the blade. In the calibration test, the static load was applied by attaching a cable to a hook on the saddle positioned at 35.05 m from the root and the blade was deformed upward by pulling the cable incrementally. Readings from all 30 strain gauges along the blade were recorded at each load increment up to the maximum load. Figure 3 shows the experimental set up for calibration pull-test. The maximum load applied was 15 kN in both the upward and downward directions to make sure the blade remains in linear elastic region. From calibration tests the relationship between the monotonic load and strain were obtained. Saddles were attached to the blade at positions that the required target BM distribution over the blade length can be applied during the fatigue testing. This was accomplished by attaching the first saddle at 30.05 m and the second saddle at 35.05 m from the blade root as shown in Figure 3c.

### 3.2. Modal Testing

In fatigue testing, the blade is resonating by setting the frequency of mass movement on the saddle equal to the first natural frequencies of the blade which is obtained from model testing. Modal testing is also used for structural health monitoring (SHM) by tracking any changes in the natural frequency of the structure caused by any damage formation. ORE Catapults standard testing procedure PR10015 [24] for modal testing was followed to obtain the frequency response function (FRF) in the flapwise direction. The transient force excitation of the blade was established by impacting at rest turbine blade once by a hammer of sufficient mass and velocity. This gives the blade an initial velocity and acceleration in the flapwise and edgewise directions. The turbine blade thus oscillates with a superposition of its discrete eigensolutions [25]. For wind turbine blades only a limited frequency band is of relevance—typically the frequency range between 0.5 Hz and 30 Hz [26].

For flapwise modes, the blade was manually excited at 38.5 m by hammer impact at the blade centre point on the pitch axis and the response of the blade was recorded by accelerometers. For edgewise modes, the blade was excited manually at the same length but impacted at the leading edge. The acceleration response signals were measured at five cross-sections along the pitch axis. Each cross section was instrumented with a uniaxial accelerometers recording the accelerations in the flapwise direction, and they were rotated for recording the acceleration in the edgewise direction. From hammer testing the first two modes of as received blade and cracked blade were determined.

### 3.3. Fatigue Testing

The 47.5 m blade was attached to Hub Centre 1, 3.75 m above the floor, with the distance from floor to roof of 25 m as shown in Figure 3a. The maximum individual force which can be applied is 600 kN, with maximum strain of 10,000 με.

The excitation device drives the saddle with a sinusoidal motion, and a closed loop control system controls the frequency and amplitude of this motion so that the strain amplitude on a selected strain gauge is maintained at the target level required in the test specification. The saddle is designed in such a way that it will be as close to the blade neutral axis as possible. Excitation devices are attached on each side of the metal case of the saddle at the Leading Edge (LE) and the Trailing Edge (TE) to excite the blade for the fatigue testing.

The target BM is the magnitude of the BM that the blade will experience in the service life. The position, magnitude of dynamic mass and amplitude of the saddles is determined to achieve the intended target BM. In the fatigue tests of as received blade, a dynamic mass of 150 kg on each side of the saddle at 30.05 m and a dynamic mass of 75 kg on each side of saddle at 35.05 m were required to produce 50% of the estimated target BM. These masses were also added to the blade during modal testing to find the natural frequencies of the blade. When the crack was extended to 1000 mm at 70% of the blade estimated target BM, the saddle resonance amplitude limit was reached. Extra masses were added to the saddles to reduce the resonance amplitude while the strain value kept unchanged.

## 4. Test Results

### 4.1. Calibration Pull-Test on as Received Blade

**Figure 4:**
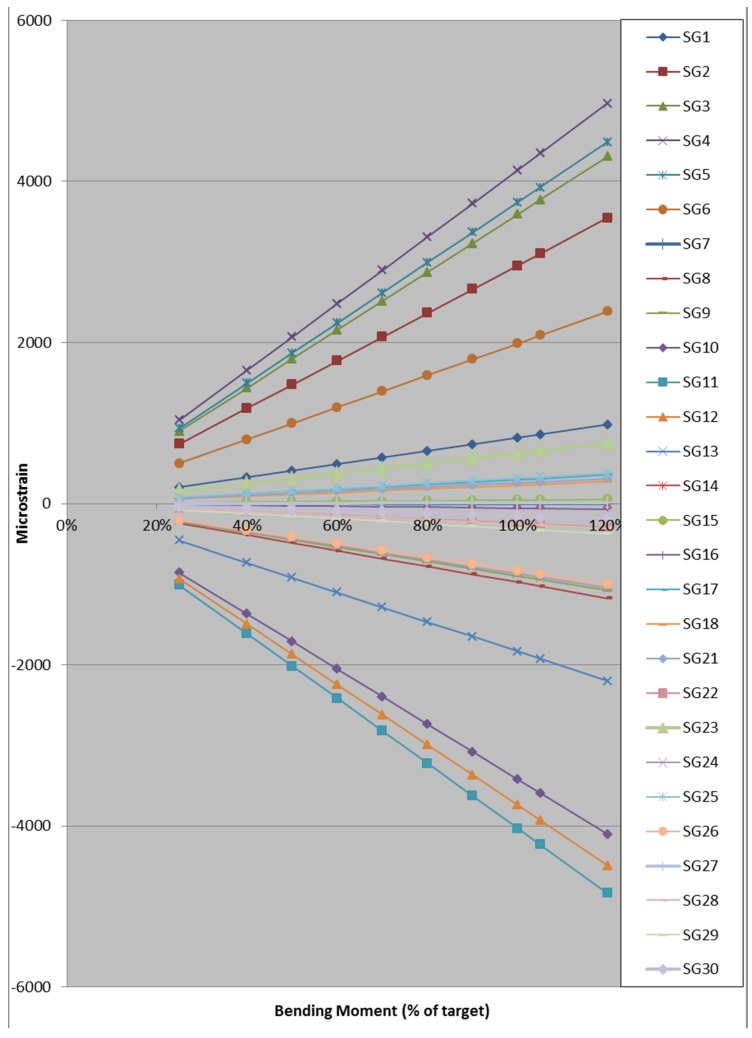
Variation of strains and root bending moment as % of estimated target BM along the as received blade.

The strains on pressure and suction side along the blade at different applied loads at 35.05 m were recorded during the calibration pull-test. The variation of the root BM with strain was calculated as shown in Figure 4. The required strain for attaining the estimated target BM in the fatigue test can be found from extrapolating the results in Figure 4. Usually the target BM should be matched but due to existing damage in the blade the maximum estimated target BM for the first two states of the blade was limited to 50% of estimated target BM. This was done to make sure the crack propagation tests could be completed without causing early stage failure of the blade. Monitoring of fatigue tests was done by controlling SG2 strain gauge readings of 1478 με at 2500 kNm BM at the root, which is equivalent to 50% of target BM.

**Figure 5:**
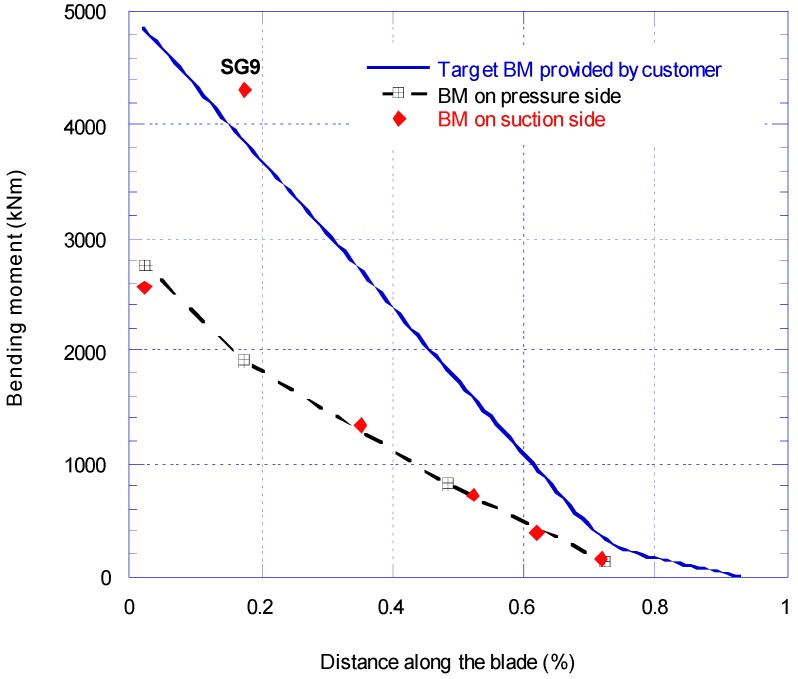
Comparison of target and applied bending moment distribution for as received blade.

Figure 5 shows the bending moment distribution along the blade. There are three set of data in Figure 5; target BM (solid line), pressure side (dash line) and suction side (diamond markers) BM. The applied BM on the blade was obtained by extrapolating the calibration test results. As explained before, the maximum target BM is set at 50% of expected BM in service. Therefore, the results for pressure and suction bending moments are at 2500 kNm at the root which is equivalent to 50% of estimated target BM. On the suction side the reading of strain gauge SG9 is unreliable and instead SG10 was used for the analysis of the bending moment.

### 4.2. Calibration Pull-Test on the Blade with 200 mm and 1000 mm Cracks

**Figure 6:**
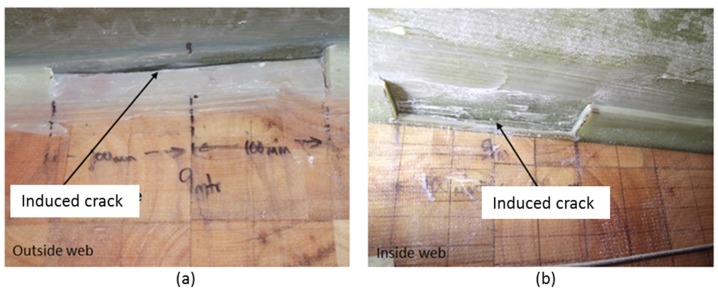
Creating a 200 mm crack at 9 m from the root. View from (**a**) outside and (**b**) inside of the shear web box.

A 200 mm crack was created by cutting the adhesive bond between the shear web and spar cap at 9 m from the blade root. Figure 6a shows the crack from outside and Figure 6b shows the crack from inside of the shear web box. Four AE sensors were fitted on the shear web wall to monitor the crack growth and to understand its behaviour during cyclic loading.

**Figure 7:**
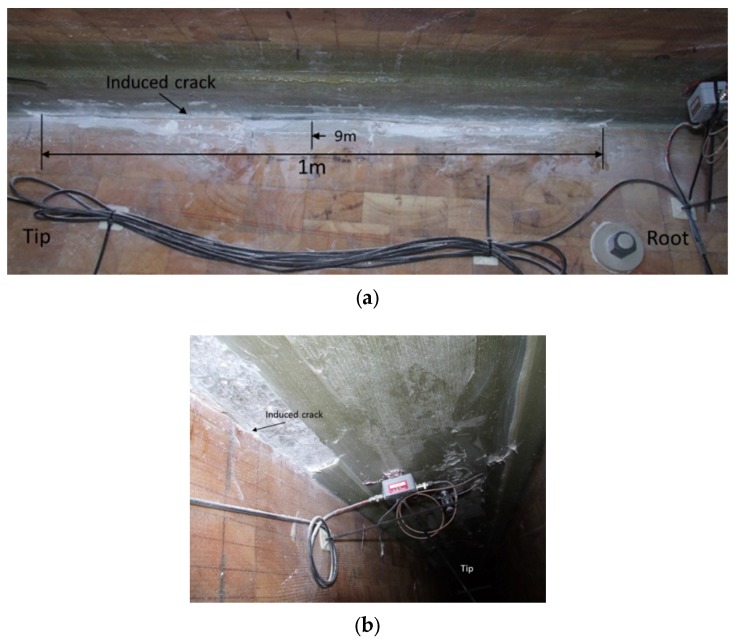
Extension of the crack to 1000 mm at 9 m from the blade root. View from (**a**) outside of the shear web box; (**b**) from inside of the shear web box.

The blade structural response to crack propagation was studied further by extending manually the 200 mm crack after calibration, modal and fatigue test to 1000 mm. The centre of de-bonded crack was kept unchanged at 9 m from the blade root and it was extended symmetrically from both sides as shown in Figure 7.

**Figure 8:**
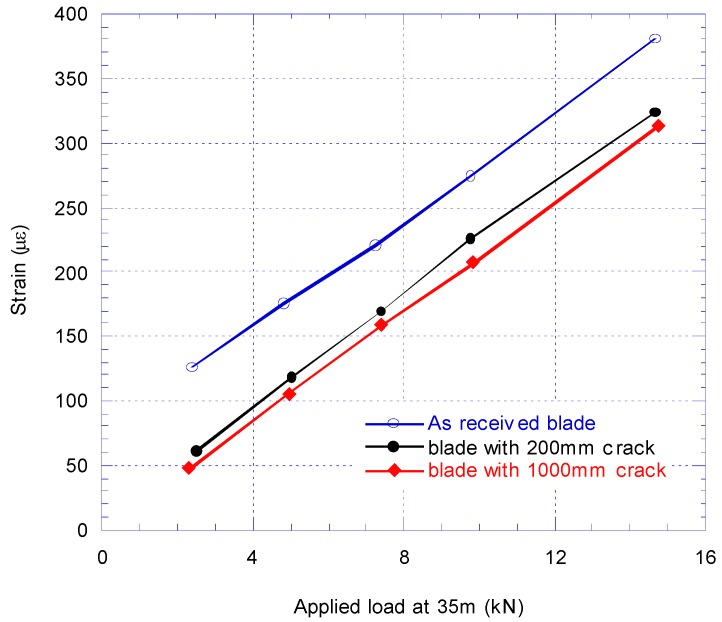
Comparison of strain at SG2 before and after introducing 200 mm and 1000 mm crack.

New calibration tests were performed to find the new bending moment distribution along the cracked blade. Figure 8 compares the calibration pull-test results from SG2 strain gauge reading for as received blade, blade with 200 mm crack and extended 1000 mm crack.

The strain gauge readings in the calibration pull-test at SG2 with debonding 200 mm of the shear web from the spar cap were decreased by about 50% of the initial load and by about 16% of the final load. The target strain at SG2 at the root became 1523 με at 2500 kNm which increased from 1478 με from before the insertion of 200 mm crack damage. When the crack damage extended further to 1000 mm, the SG2 strain gauge reading was decreased by 64% at the initial load while at the final load, the SG2 reading decreased by about 20% (see Figure 8).

**Figure 9:**
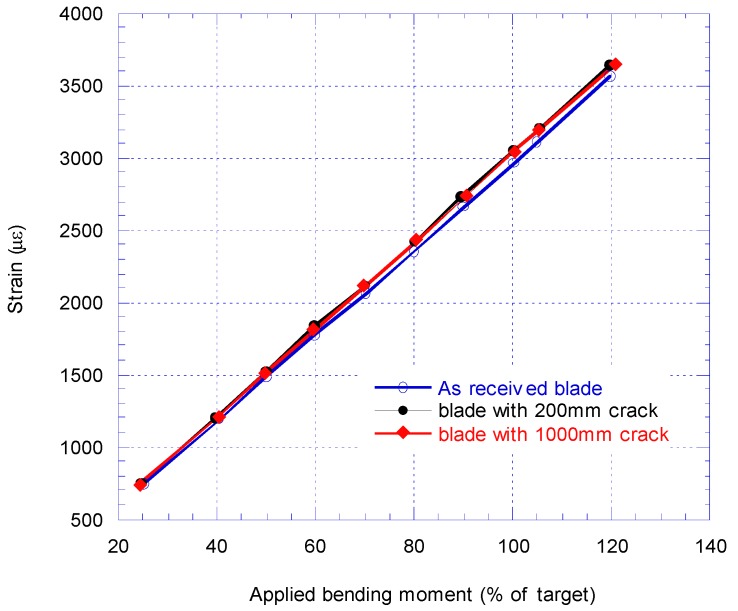
Comparison of bending moment from SG2 reading for three cases of the blade.

Figure 9 compares the BM at the blade root (as % of target BM) versus strain at SG2 for as received blade, and when 200 mm and 1000 mm crack introduced to the blade. At 70% BM, SG2 reaches 2132 με for an induced crack of 200 mm while for the 1000 mm extended crack the SG2 reading is 2135 με. The slight difference could be due to a number of reasons such as added saddle mass, movement of the saddle, ±0.5% accuracy of strain gauges, temperature, etc.

Note that in calibration pull-test with crack of 1000 mm, the mass on either side of the saddle at 30.05 m was increased from 150 kg to 200 kg, and at 35.05 m, the mass was increased from 75 kg to 100 kg, to become similar to the fatigue test set up.

**Figure 10:**
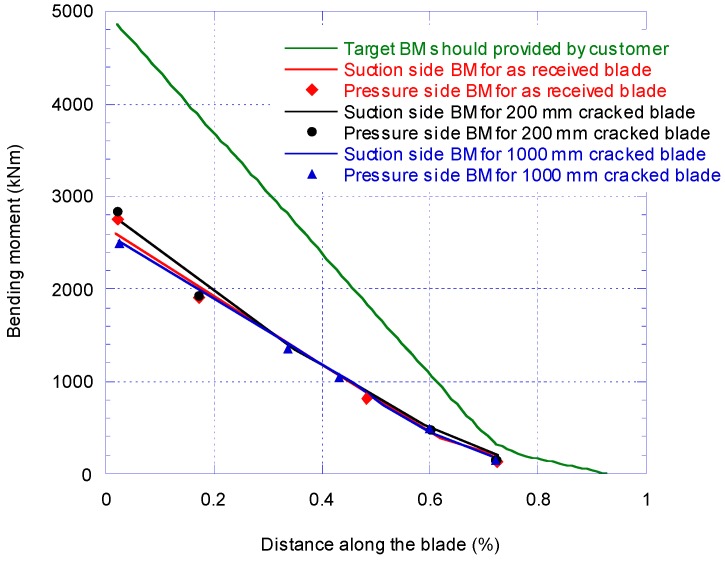
Comparison of target and measured BM distribution with crack.

Figure 10 compares the BM distribution along the blade for all three calibrations pull-test when the maximum BM at the root is set equal to 50% of the maximum target BM at the root. The result for the pressure side BM is lower than the suction side. When comparing the results of the 1000 mm cracked blade at 10% of the blade length, the strain gauge reading decreased by 13.1% in comparison with the 200 mm crack damage.

### 4.3. Modal Test on the Blade

#### 4.3.1. Modal Test on as Received Blade

**Figure 11:**
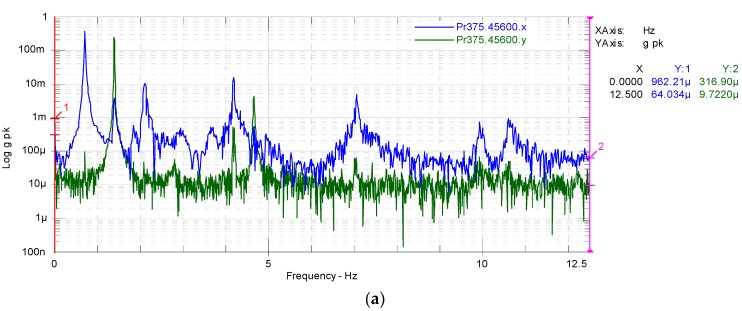

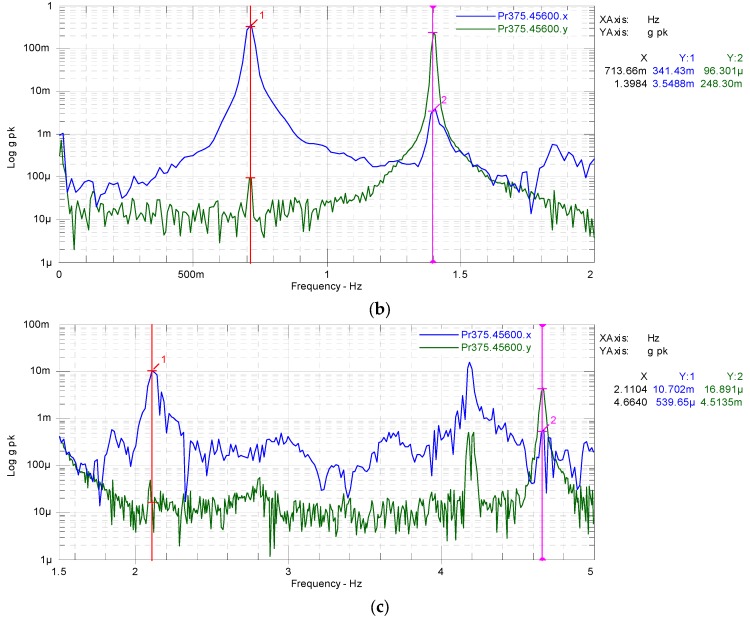
Frequency response function for as received blade without saddles in flapwise (blue line) and edgewise (green line) directions: (**a**) an overview; (**b**) the first modes and (**c**) the second.

The results of modal test for the as received blade are shown in Figure 11a where the blue line is the result of flapwise direction, and green line is the result of edgewise direction.

Figure 11b shows a magnified area of Figure 11a between 0 Hz and 2 Hz to quantify accurately the results for the first natural frequency. The first natural frequency for flapwise (blue line) and edgewise (green line) were 0.713 Hz and 1.398 Hz, respectively.

Figure 11c shows a magnified area of Figure 11a between 1.5 Hz and 5 Hz where the second mode shape occurs. The second natural frequencies for flapwise and edgewise were at 2.1 Hz and 4.66 Hz, respectively. The modal test of as received blade with saddles attached at 30.05 m and 35.05 m were also carried out and the first natural frequency was at 0.562 Hz for flapwise mode, a reduction of about 21.2% from 0.713 Hz.

#### 4.3.2. Modal Test on the Blade with Crack of 200 mm and 1000 mm

The modal testing for flapwise directions of blade with 200 mm and 1000 mm crack, together with the as received blade with attached saddles has been done. No changes in the natural frequencies were noticed and the first mode remained at 0.562 Hz.

For the blade with the 1000 mm crack, the mass on either side of the saddle at 30.05 m was increased from 150 kg to 200 kg and at 35.05 m the mass was increased from 75 kg to 100 kg to reduce the stroke amplitude of masses which were exceeded the limit. The resultant modal test showed a slight shift in the first natural frequency from 0.562 Hz to 0.556 Hz which is to be expected.

In summary the first natural frequency of as received blade, the blade with 200 mm and 1000 mm crack remained at 0.562 Hz. However, the first natural frequency for the blade with 1000 mm crack and with added mass was reduced slightly to 0.556 Hz.

## 5. Fatigue Tests

The blade was excited by two saddles sitting at 30.05 m and 35.05 m from the root of the blade as shown in Figure 3. The saddles masses oscillated at just below the determined first natural frequency of the blade to excite the blade to reach to the specific strain at a preselected strain gauge SG2 located at 8 m from the root on pressure side in longitudinal direction.

### 5.1. Fatigue Test Results before Crack Insertion

The test conditions and fatigue test results for as received blade are summarised in Table 3 where the target strain at SG2 is set at 1478 με, reaching 2500 kNm, 50% of target BM at the root as predicted from the calibration pull-test described in Section 4.1. After 5241 cycles a visual inspection of the blade was performed and three new cracks were detected. The first cracks appeared at 34 m in the TE where a 450 mm adhesive joint failure occurred. The second crack of a length of 300 mm occurred at the adhesive joint in the TE at 31 m from the root. The third crack formed at the adhesive joint in the LE at 28.36 m.

In the test, the acoustic emission (AE) sensors recognised damage around the same regions as they were found by visual inspection. After 58,665 cycles the blade has been inspected visually and no new crack was found, nor did the existing crack propagate. The fatigue test on as received blade was stopped after 65,217 cycles and at the end of the test, the original cracks had not propagated further. It is likely that an edgewise test rather than a flapwise test would have been more effective at growing the crack because the spar cap adhesive bond would experience greater shear stresses during an edgewise test.

**Figure 12:**
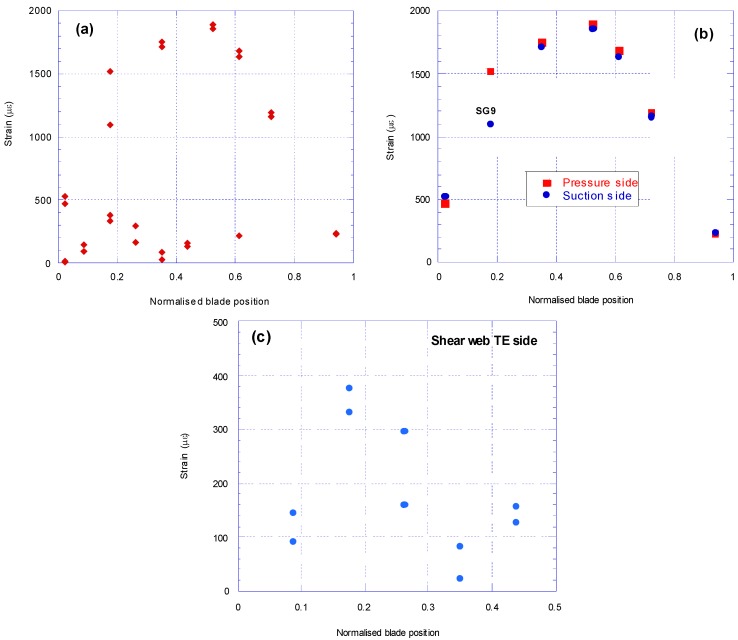
Strains along the as received blade at the first cycle of fatigue test: (**a**) all 30 strain gauges; (**b**) only for gauges on pressure and suction side and (**c**) only for gauges on the shear web on TE side.

The strains at the first cycle along the as received blade from 30 strain gauges are shown in Figure 12a. The highest strains are experienced between 35% and 65% of the blade length from the blade root.

Strain gauge results on the skin of the pressure and suction sides at the first cycle are shown in Figure 12(b), where the strain along the blade varies between 236 με and 1891 με. The highest strain on shell skin is at 52% of the overall length. The results are almost identical on the pressure side and suction side, apart at the 17% blade location where the pressure side shows a reading of 423 με more than the suction side. This indicated that the strain gauge SG9 on the pressure side was faulty and therefore its reading was discarded.

Strain gauges SG21 to SG30 are attached to the shear web on TE side at ±45° and located from 4 m to 20 m from the root. The readings from these gauges at the first cycle are shown in Figure 12c. The strain along the first half of the blade length from the root varies between 24.5 με and 378 με. In flapwise fatigue, the maximum strain readings in the shear web occurred at SG23 and SG24 attached to the shear web, located at 17.5% of the blade length from the root.

### 5.2. Fatigue Test Results for Blade with 200 mm Crack

**Figure 13:**
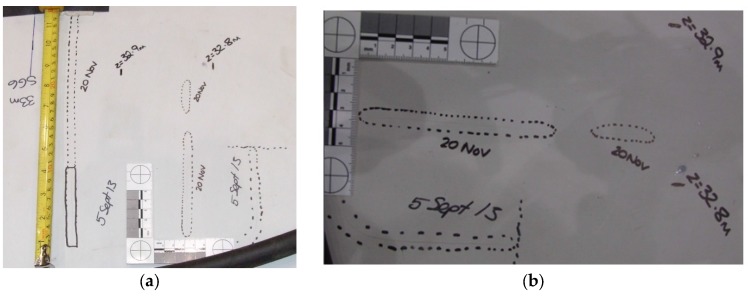
(**a**) Propagation of existing crack at 32.96 m after 26,851 cyclic loading; (**b**) Formation of hair line cracks at 32.8 m after 26,851 cyclic loading.

The test conditions and results for the blade with a 200 mm crack are shown in A pre-existing crack at around 32.96 m had extended by 190 mm in the middle of the blade as shown in Figure 13a with the overview image of the two cracks found. Figure 13b shows a close inspection of the new cracks found at about 32.83 m from the blade root which propagated for 110 mm and 40 mm on the middle cross section at this point. The next visual inspection was carried out after 39,934 cycles, where the applied BM value had been increased to 70% of the maximum target BM. The visual inspection showed an existing crack at about 32.96 m propagated 15 mm along the blade longitudinal direction at the TE midpoint. Finally, a visual inspection was made after 62,110 cycles and no further crack formation or propagation was observed and the induced crack stayed unchanged at its original length.

Table 4. Initially, a 2500 kNm bending moment at the root was applied which resulted in 1523 με at SG2. The strain at this location had increased from 1478 με before the insertion of crack, showing a 3% increase. A visual inspection was performed after 8063 cycles to check if any existing cracks had propagated or new ones formed. No changes to the existing cracks or to the 200 mm crack at 9 m distance were observed. Later on the visual inspection was repeated after 26,851 cycles. A pre-existing crack at around 32.96 m had extended by 190 mm in the middle of the blade as shown in Figure 13a with the overview image of the two cracks found. Figure 13b shows a close inspection of the new cracks found at about 32.83 m from the blade root which propagated for 110 mm and 40 mm on the middle cross section at this point. The next visual inspection was carried out after 39,934 cycles, where the applied BM value had been increased to 70% of the maximum target BM. The visual inspection showed an existing crack at about 32.96 m propagated 15 mm along the blade longitudinal direction at the TE midpoint. Finally, a visual inspection was made after 62,110 cycles and no further crack formation or propagation was observed and the induced crack stayed unchanged at its original length.

### 5.3. Fatigue Test Results for Blade after Extending the Crack to 1000 mm

The test conditions and results for the blade after extending the inserted crack to 1000 mm are shown in Table 5. Initially 3500 kNm bending moment equivalent to 70% of target BM were applied at the root which resulted in 2135 με at SG2. The strain at this location remained nearly unchanged close to 2132 με before the crack extended from 200 mm to 1000 mm. Visual inspections of the blade were performed after 3967; 16,781 and 18,764 cycles to check the state of the cracks in the blade. No changes to the existing cracks or to the induced crack at 9 m were detected.

The load was then increased to 90%, 100%, 105% and 110% of the blades estimated target BM value. For these tests the blade first mode natural frequency was obtained using ORE software rather than doing modal tests to save time.

As mentioned earlier, for this state of the blade mass has been added to the saddles to reduce the saddle stroke amplitude. The mass on the saddle at 30.05 m was increased from 150 kg to 200 kg and at 35.05 m was increased from 75 kg to 100 kg on both sides of the saddle, to achieve 70% of the nominal target BM. At 90% of the nominal BM loading, the mass at 30.05 m was increased to 325 kg and at 35.05 m increased to 150 kg on both sides of the saddle. Finally, at 100% and 110% of the nominal BM loading, the mass at 30.05 m was increased to 400 kg and at 35.05 m increased to 200 kg on both sides of the saddle.

**Figure 14:**
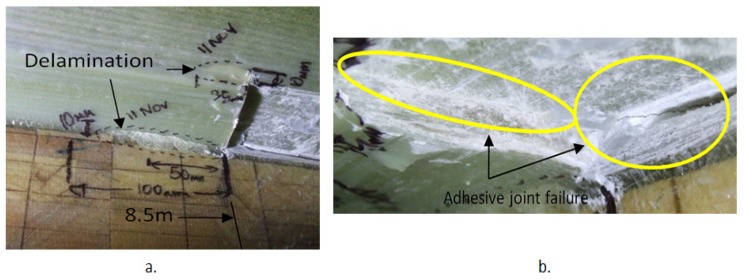

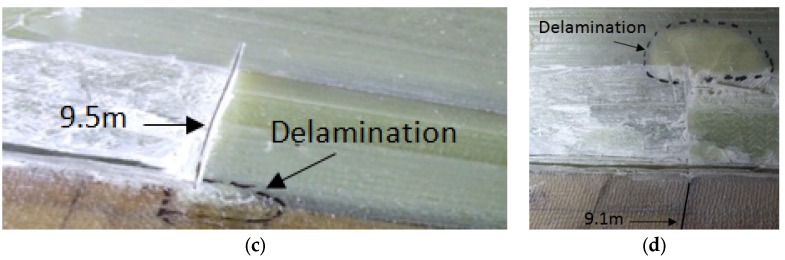
Damaged areas around the root: (**a**) delamination at 8.5 m (**b**) adhesive joint failure at 8.5 m; (**c**) delamination inside the web box at 9.5 m and (**d**) delamination inside the web box at 9.1 m.

A visual inspection of the blade after 34,776 cycles showed a number of cracks was formed around the 1000 mm extended crack. Inside the web box at 8.5 m from the root, two delaminated areas each at 10 mm in width were detected. The delamination connected to the web had a delamination length of 83 mm and the delamination furthest away from the web had a length of 35 mm as shown in Figure 14a. At 8.5 m, the induced crack through the adhesive joint had propagated from its corner and extended through the plies causing delamination on the web lip, indicating that the crack plane has been deviated from its original path; see Figure 14b. At 9.5 m from the blade root delamination had occurred at the corner of the 1000 mm crack as shown in Figure 14c. Figure 14d shows delamination at 9.1 m at the location of the corner of the 200 mm crack. The fact that the crack bridged across from the unreinforced adhesive to shear web laminate is interesting, and is probably a result of the complex mode II loading that would be induced by a flapwise test in this region. It is also possible that when cutting the crack the blade had travelled into reinforced material as well as through the adhesive, which would make the bridging more likely to occur.

**Figure 15:**
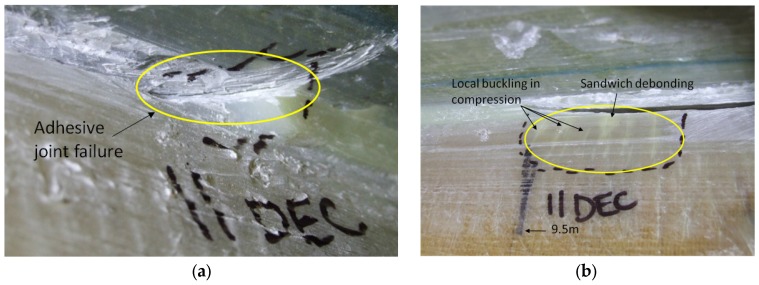
(**a**) Adhesive debonding outside the web box at 8.5 m; (**b**) local buckling under compression and sandwich debonding outside the web box at 9.5 m.

The adhesive joint had debonded outside the web box at 8.5 m as shown in Figure 15a indicating that the crack had propagated all the way through the web. At 9.5 m the blade experienced multiple local buckling under compression, and debonding in the sandwich propagated into the web, as shown in Figure 15b.

A visual inspection of the blade after 47,206 cycles showed a number of further damages around the 1000 mm extended crack. At 8.5 m inside the web box, there were two areas of delamination each 10 mm in width. The delamination connected to the web, where the delamination length had extended by 25 mm. At 8.9 m inside the web box, delamination had occurred at the corner of the original 200 mm induced crack. Inside the web box at 9.5 m, delamination had extended from the corner of the 1000 crack to the lip of the web.

**Figure 16:**
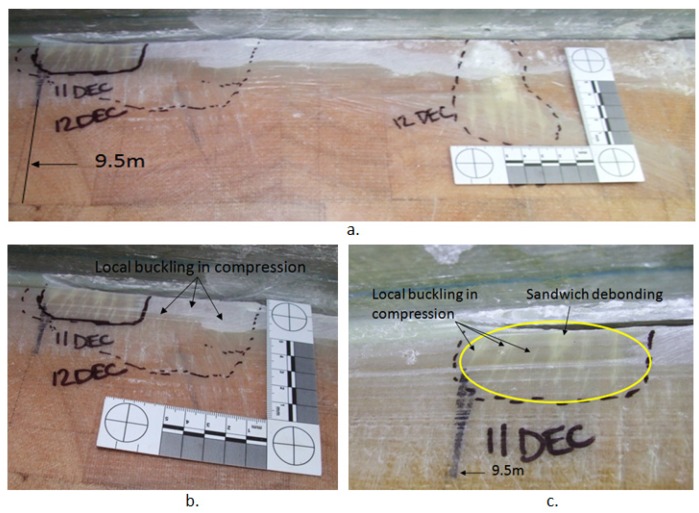
Outside web at 9.5 m (**a**) overview of local buckling under compression and sandwich debonding (**b**) magnified view of damage at 9.5 m (**c**) magnified view of damage at 9.3 m.

Figure 16a shows outside the web box at 9.5 m with an overview of the damaged areas. The blade experienced multiple local buckling under compression, and sandwich debonding going into the web. Figure 16b is the magnified view of the damaged area at 9.5 m and Figure 16c shows the magnified view of the damaged area at 9.3 m. The crack had also propagated 100 mm in the adhesive joint at 8.5 m.

**Figure 17:**
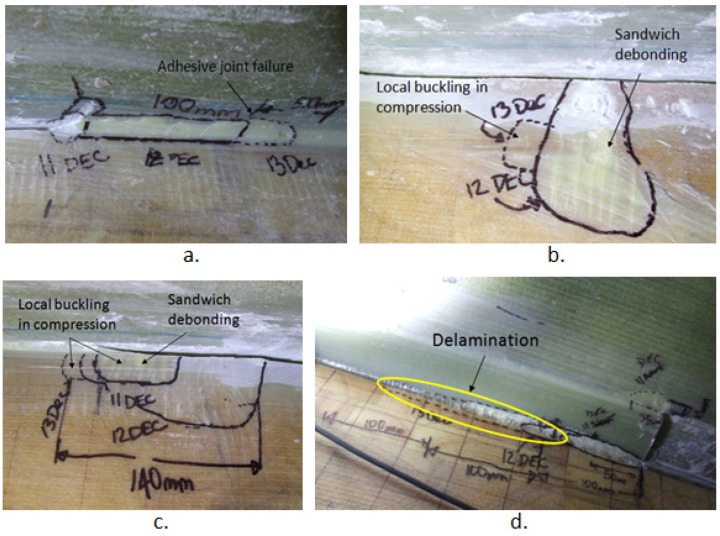
Crack propagation within the web; (**a**) adhesive joint failure outside the web; (**b**,**c**) local buckling under compression outside the web; (**d**) delamination inside the web.

A visual inspection of the blade after 56,762 cycles showed that many of the cracks had extended along the web within the 1000 mm crack area as illustrated in Figure 17. At 9.5 m inside the web some delamination had also begun to occur.

**Figure 18:**
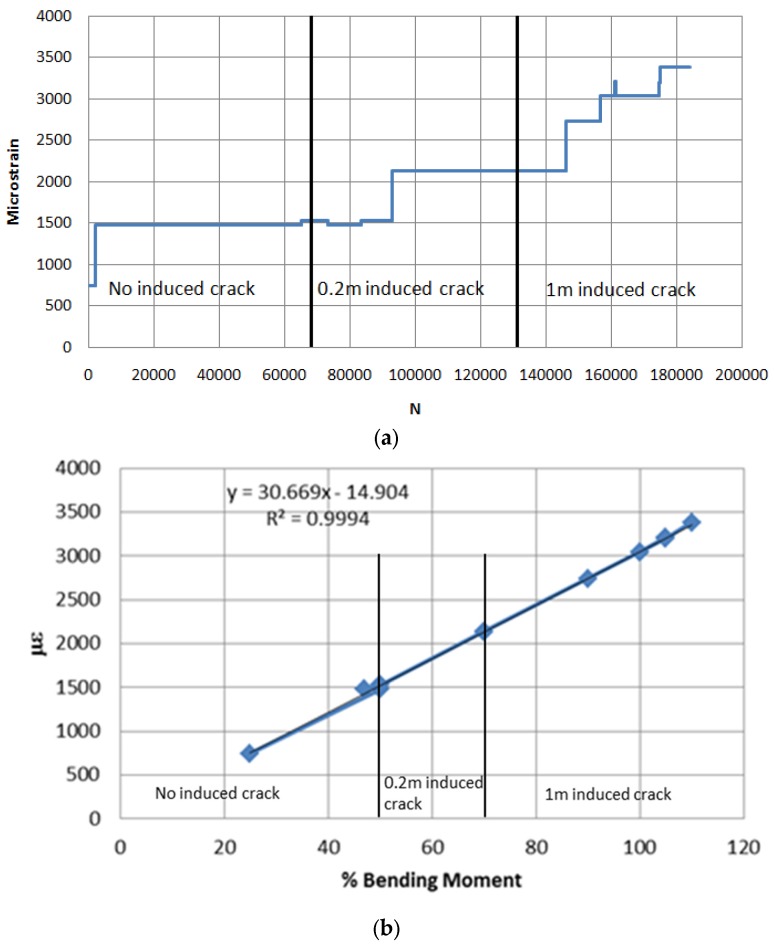
(**a**) SG2 reading versus number of cycles throughout fatigue testing and (**b**) Variation of SG2 strain versus BM loading throughout all three stages of fatigue testing.

The variation of SG2 strain versus number of cycles and percentage of applied BM experienced by the blade at all stages of the fatigue testing for three states of the blade are shown in Figure 18. The figure shows that no nonlinearity occurred during the entire testing cycles, and that the various damages created during the fatigue tests had not yet caused any noticeable deterioration to the structural stiffness of the blade.

## 6. Conclusions

The blades are one of the most critical components of the wind turbine. The structural integrity of the whole wind turbine depends on the blade, and therefore they have to be tested in order to ensure that the blade can withstand both the ultimate loads and the fatigue loads to which the blade will be subjected during its entire service life. Testing of the wind turbine blades in static and fatigue loading reveal any possible weakness in the design, materials and the manufacturing processes. Removing the weakness will save both the expensive cost of maintenance or replacement.

In this paper, all required steps for full-scale fatigue testing of a 47.5 m blade have been discussed. The starting point was acquiring the required information for fatigue from the calibration pull-tests. From this test the magnitude of strain for monitoring the target BM at the root of the blade in fatigue testing has been identified. These tests were performed in three stages of blade: (i) as received blade; (ii) when a 200 mm crack was introduced and (iii) when the crack extended to 1000 mm. From the calibration pull-tests the strain-bending moment relationship of the blade has been identified according to the target BM which the blade expected to experience in its service life. These data were used for monitoring the applied bending moment in the fatigue tests.

For fatigue testing the first natural frequency of the blade is required. The blade natural frequencies over a range of frequency domain were found by modal testing using hammer impact. The modal testing has been done in both flapwise and edgewise directions for all three states of the blade while the masses were attached to the saddles. The measured natural frequency of the first mode of as received blade was 0.562 Hz. This frequency has not changed when the 200 mm crack was introduced in the blade and when the crack further extended to 1000 mm. However, the first natural frequency had changed slightly by 0.006 Hz for 1000 mm crack when extra mass was added to the saddles.

Before start of calibration and fatigue testing, the blade had two areas of defects at 4% and 71% of blade length. After 184,089 cyclic loading of the blade, the areas that experienced most damage and fracture were between 62% and 74% of the blade length from the root, but no damage was found within the shear web.

The sensitivity of the blade structure to damage under cyclic loading was investigated by introducing a 200 mm crack at 9 m from the root. The crack was created by debonding the web from the spar cap. The crack did not propagate in fatigue loading up to 50% of the estimated target BM. When the load increased to 70% of target BM, some damages have been detected on the pressure side of the blade. However, the 200 mm crack did not propagate under these conditions up to 62,110 cycles.

Finally, the 200 mm crack at 9 m from the root has been extended to 1000 mm. The crack began to propagate when the applied load exceeded 100% estimated target BM. The blade experienced delamination around the crack tip of the 1000 mm crack at the web, adhesive joint failure occurred on the outside of the web, compression failure and sandwich debonding occurred on the inside of the web, and delaminations were visible on the web just below the 1000 mm crack. The test was being conducted with only flapwise loading being applied—it would have been interesting to investigate the rate of crack propagation under edgewise loading as well.

As the blade design was not available, it was impossible to model which crack size would be likely to grow. Therefore, it is not possible to draw any conclusions about whether it is safe to continue operating with a crack of less than 200 mm length in the spar cap—shear web bond. Also, because the loads were not at 100% until the blade had a 1000 mm crack, the load magnitude will of course have been a factor in the increased rate of crack propagation of the larger crack.

## Figures and Tables

**Table 1:** Locations and position of strain gauges on the blade.

Gauge ID	Distance from Root (m)	LW or WW	LE or TE	Edge Distance (mm)	Long. or Trans.	Internal or External
SG1	1.0	P	-	-	Long 0	Ext
SG2	8.0	P	-	-	Long 0	Ext
SG3	16.0	P	-	-	Long 0	Ext
SG4	24.0	P	-	-	Long 0	Ext
SG5	28.0	P	-	-	Long 0	Ext
SG6	33.0	P	-	-	Long 0	Ext
SG7	43.0	P	-	-	Long 0	Ext
SG8	1.0	S	-	-	Long 0	Ext
SG9	8.0	S	-	-	Long 0	Ext
SG10	16.0	S	-	-	Long 0	Ext
SG11	24.0	S	-	-	Long 0	Ext
SG12	28.0	S	-	-	Long 0	Ext
SG13	33.0	S	-	-	Long 0	Ext
SG14	43.0	S	-	-	Long 0	Ext
SG15	1.0	-	LE	0	Long 0	Ext
SG16	1.0	(p)	TE	0	Long 0	Ext
SG17	16.0	(p)	TE	50	Long 0	Ext
SG18	28.0	(p)	TE	50	Long 0	Ext
SG21	4.0	Shear web TE, mid			+45	Int
SG22	4.0	Shear web TE, mid			−45	
SG23	8.0	Shear web TE, mid			+45	Int
SG24	8.0	Shear web TE, mid			−45	
SG25	12.0	Shear web TE, mid			+45	Int
SG26	12.0	Shear web TE, mid			−45	
SG27	16.0	Shear web TE, mid			+45	Int
SG28	16.0	Shear web TE, mid			−45	
SG29	20.0	Shear web TE, mid			+45	Int
SG30	20.0	Shear web TE, mid			−45	

P = Pressure side, S = Suction side, LW = Leeward, WW = windward.

**Table 2:** Accelerometers location.

Accelerometer Number	Distance from Root (m)
1	9
2	18
3	27
4	36
5	45.7 (tip)

**Table 3:** Fatigue test results for the as received blade.

% of BM Nominal Value	Cycles ±200	Cumulative Cycles	Strain at SG2 (με) ±0.5%	Frequency (Hz)	Crack Distance from Root (m)	Crack Length (mm)	Location	Amplitude of Resonance Mass (mm)
25	2217	2217	740	0.43	-	-	-	77
50	3024	5241	1478	0.56	28.36	640	LE	154
					31	300	TE	
					34	450	TE	
50	12,096	17,337	1478	0.56	-	-	-	154
50	14,112	31,449	1478	0.56	-	-	-	154
50	14,112	45,561	1478	0.56	-	-	-	154
50	13,104	58,665	1478	0.56	-	-	-	154
50	6552	65,217	1478	0.56	-	-	-	154

**Table 4:** Fatigue test condition and results for the blade with a 200 mm crack.

% of BM Nominal Value	Cycles ±200	Cumulative Cycles	Strain at SG2 (με) ±0.5%	Frequency (Hz)	Crack Distance from Root (m)	Crack Length (mm)	Location	Amplitude of Resonance Mass (mm)
50	2923	2923	1523	0.56	-	-	-	158
50	5140	8063	1523	0.56	-	-	-	158
47	10,281	18,344	1478	0.56	-	-	-	154
50	8507	26,851	1523	0.56	≈32.96	190	TE-Mid	158
					≈32.83	110	TE-Mid	
					≈32.83	40	TE-Mid	
50	806	27,657	1523	0.56	-	-	-	158
70	12,277	39,934	2132	0.56	≈32.96	15	TE-Mid	260
70	12,096	52,030	2132	0.56	-	-	-	260
70	10,080	62,110	2132	0.56	-	-	-	260

**Table 5:** Fatigue test results for blade with extended crack to 1000 mm.

% of bm Nominal Value	Cycles ±200	Cumulative Cycles	Strain at SG2 (με) ±0.5%	Frequency (Hz)	Crack Distance from Root (m)	Crack Length (mm)	Location	Amplitude of Resonance Mass (mm)
70	3967	3967	2130	0.551	-	-	-	219
70	12,814	16,781	2135	0.551	-	-	-	219
70	1983	18,764	2135	0.551	-	-	-	219
90	5970	24,734	2737	0.535	-	-	-	234
90	4603	29,337	2737	0.535	-	-	-	234
100	4451	33,788	3040	0.524	-	-	-	230
105	94	33,882	3211	0.524	-	-	-	250
100	894	34,776	3040	0.497	8.5	35.83	IW *, OW	174
					9.2	-	IW	
					9.5	-	IW, OW	
100	12,430	47,206	3040	0.497	9.5	DD **	IW, OW	174
					9.1	DD	IW	
					8.5	DD	IW, OW	
105	357	47,563	3201	0.497	-	DD	-	220 at 30.05 m
								165 at 35.05 m
110	9199	56,762	3380	4962	8.5	DD	IW, OW	220 at 30.05 m
					9.5	DD	IW, OW	165 at 35.05 m

* IW = Inside the web, OW = Outside the web, ** DD = Detailed discussion in the report.

## References

[1] Malhotra P., Hyers R., Manwel J., McGowan J. (2012). A review and design study of blade testing systems for utility-scale wind turbines. Renew. Sustain. Energy Rev..

[2] Yang B., Sun D. (2013). Testing, inspecting and monitoring technologies for wind turbine blades: A survey. Renew. Sustain. Energy Rev..

[3] Al-Khudairi O., Hadavinia H., Lewis E., Osborne B., Bryars L.S., Elmarakbi A. (2014). Building Delamination Fracture Envelope under Mode I/Mode II Loading for FRP Composite Materials. Advanced Composite Materials for Automotive Applications: Structural Integrity and Crashworthiness.

[4] Cadavid M.O., Al-Khudairi O., Hadavinia H., Goodwin D., Liaghat G.H. (2017). Experimental studies of stiffness degradation and dissipated energy in glass fibre reinforced polymer composite under fatigue loading. Polym. Polym. Compos..

[5] Al-Khudairi O., Hadavinia H., Waggott A., Lewis E., Little C. (2015). Characterising mode I/mode II fatigue delamination growth in unidirectional fibre reinforced polymer laminates. Mater. Des..

[6] (2014). IEC 61400-23:2014: Wind Turbines—Part 23: Full-Scale Structural Testing of Rotor Blades.

[7] (2001). IEC TS 61400-23:2001: Wind Turbine Generator Systems—Part 23: Full-Scale Structural Testing of Rotor Blades.

[8] Vertias D.N. Standard DNV-DS-J102: Design and Manufacture of Wind Turbine Blades, Offshore and Onshore Wind Turbines. http://rules.dnvgl.com/docs/pdf/DNV/codes/docs/2010-11/DS-J102.pdf.

[9] (2006). BS EN 61400-1:2005+A1:2010: Wind Turbines—Design Requirements.

[10] (2010). Rules and Guidelines IV Industrial Services 1 Guideline for the Certification of Wind Turbines.

[11] (2005). Rules and Guidelines IV Industrial Services 2 Guideline for the Certification of Offshore Wind Turbines.

[12] Yang J., Peng C., Xiao J., Zeng J., Xing S., Jin J., Deng H. (2013). Structural investigation of composite wind turbine blade considering structural collapse in full-scale static tests. Compos. Struct..

[13] Kühlmeier L. (2007). Buckling of Wind Turbine Rotor Blades. Analysis, Design and Experimental Validation. Ph.D. Thesis.

[14] Lee H.G., Park J. (2016). Static test until structural collapse after fatigue testing of a full-scale wind turbine blade. Compos. Struct..

[15] Jensen F.M., Falzon B.G., Ankersen J., Stang H. (2006). Structural testing and numerical simulation of 34 m composite wind turbine blade. Compos. Struct..

[16] Cox K.A. (2014). Echtermeyer, Effects of composite fiber orientation on wind turbine blade buckling resistance. Wind Energy.

[17] Lee H.G., Kang M.G., Park J. (2015). Fatigue failure of a composite wind turbine blade at its root end. Compos. Struct..

[18] Mishnaevsky L., Dai G. (2014). Hybrid and hierarchical nanoreinforced polymer composites: Computational modelling of structure-properties relationships. Compos. Struct..

[19] Domun N., Hadavinia H., Zhang T., Sainsbury T., Liaghat G.H., Vahid S. (2015). Improving fracture toughness and strength of epoxy using nanomaterials—A review of current status. Nanoscale.

[20] Domun N., Hadavinia H., Zhang T., Liaghat G.H., Vahid S., Paton K., Spacie C., Sainsbury T. (2017). Improving the fracture toughness properties of epoxy using graphene nanoplatelets at low filler content. Nanocomposites.

[21] Thomsen O. (2009). Sandwich materials for wind turbine blade—Present and future. J. Sandw. Struct. Mater..

[22] Buckney N., Green S., Pirrera A., Weaver P.M. (2013). On the structural topology of wind turbine blades. Wind Energy.

[23] Sørensen B.F., Jørgensen E., Debel C.P., Jensen F.M., Jensen H.M., Jacobsen T.K., Halling K.M. Improved Design of Large Wind Turbine Blade of Fibre Composites Based on Studies of Scale Effects (Phase 1)—Summary Report. http://orbit.dtu.dk/fedora/objects/orbit:90493/datastreams/file_7702048/content.

[24] ORE’s Standard Testing Procedure for Modal Testing PR10015.

[25] Larsen G.C., Kretz A. (1995). Experimental Determination of Stiffness Distributions and Mode Shapes of Wind Turbine Blades.

[26] Larsen G.C., Hansen M., Baumgart A., Carlén I. Modal Analysis of Wind Turbine Blades. http://orbit.dtu.dk/fedora/objects/orbit:90910/datastreams/file_7712483/content.

